# On the Dimension of the Singular Set of Perimeter Minimizers in Spaces with a Two-Sided Bound on the Ricci Curvature

**DOI:** 10.1007/s12220-024-01784-6

**Published:** 2024-10-23

**Authors:** Alessandro Cucinotta, Francesco Fiorani

**Affiliations:** Mathematical Institute, University of Oxford, Oxford, UK

**Keywords:** Perimeter minimizing sets, Ricci curvature, Analysis of singularities, 49Q05, 58J90, 53C23

## Abstract

We show that the Hausdorff dimension of the singular set of perimeter minimizers in noncollapsed limits of manifolds with two-sided bounds on the Ricci curvature is at most $n-5$, where *n* is the dimension of the ambient space. The estimate is sharp.

## Introduction

We consider noncollapsed limits of manifolds with two-sided bounds on the Ricci curvature. These are pointed metric spaces (*X*, *d*, *p*) arising as pointed Gromov-Hausdorff limits of sequences of pointed Riemannian manifolds $(M^n_k,d_k,x_k)$, where $d_k$ denotes the Riemannian distance, satisfying

$$\begin{aligned} |\textrm{Ric}_{M^n_k}| \le n-1, \quad \text {and} \quad \textrm{vol}(B_1(x_k)) \ge v > 0 \quad \text {for every } k. \end{aligned}$$

A non-exhaustive list of works where spaces satisfying the conditions above were initially studied is [11, 12, 16, 23, 46]. The study of metric spaces arising as Gromov-Hausdorff limits of Riemannian manifolds with a uniform lower Ricci curvature bound was carried out in [24–26], among others.

We are concerned with perimeter minimizing sets in noncollapsed limits of manifolds with two-sided bounds on the Ricci curvature. The notion of perimeter in metric measure spaces was studied in [2, 4, 5, 7, 42], among others. In recent years, the theory of sets of finite perimeter was further studied in [6, 19, 20] (among others) in the setting of metric measure spaces with a synthetic notion of Ricci curvature lower bounds, known as RCD spaces. The Riemannian Curvature Dimension condition RCD$(K, \infty )$ was introduced in [9] (see also [8, 38]) while its finite dimensional counterpart RCD(K,n) was formalized in [38]. For a thorough introduction to the topic we refer to the survey [3] and the references therein.

Some fundamental steps towards understanding perimeter minimizing sets in the context of RCD spaces and Ricci limit spaces were carried out in [44] and [34, 35] respectively. Other properties were then investigated in [29, 30, 37]. Let us point out that minimal hypersurfaces in Riemannian manifolds are locally boundaries of locally perimeter minimizing sets. Moreover, the study of sets of finite perimeter in the RCD setting, due to their convergence and stability properties, allows to deduce new results about classical area minimizing hypersurfaces and isoperimetric sets in Riemannian manifolds (see, for instance, [14, 15, 30, 44]).

One of the key advances in the study of perimeter minimizing sets in Euclidean spaces was understanding the Hausdorff dimension of their singular set. A fundamental result obtained by De Giorgi [31] and refined by Federer following the work of Simons shows that a perimeter minimizing set $E \subset \mathbb {R}^n$ is smooth outside a closed set of Hausdorff dimension at most $n-8$. The regularity of perimeter minimizing sets in RCD spaces was studied in [44]. To report here the relevant result, we introduce some notation.

A *metric measure space* is a triple $(X,d,\mathfrak {m})$ such that (*X*, *d*) is a metric space and $\mathfrak {m}$ is a Radon measure on *X*. We denote by $\mathcal {H}^n$ the *n*-dimensional Hausdorff measure on (*X*, *d*). Given an RCD(K,n) space $(X,d,\mathcal {H}^n)$, we denote the set of regular points of *X* as $\mathcal {R}(X)$ (see the comment after Definition 2.12). The regular and singular sets of a locally perimeter minimizing set $E \subset X$ (see Definition 2.14) are denoted respectively by $\mathcal {R}^E$ and $\mathcal {S}^E$ (see Definition 2.16). We denote by $\mathcal {S}_{k}$ and $\mathcal {S}^E_{k}$ respectively the *k*-dimensional singular stratum of *X* and *E* (see Definitions 2.4 and 2.17). The space *X* is said to be without boundary if $\mathcal {S}_{n-2} {\setminus } \mathcal {S}_{n-1} = \emptyset$.

We highlight that a point $x \in \partial E$ is regular if it is a regular point for the ambient space *X*, and additionally the tangent of *E* at *x* is a half space. This definition is slightly different from the classical one in the Euclidean setting: if one exploits the non-smooth definition in the smooth setting, the usual uniqueness requirement for the tangent space needs to be dropped, as different half spaces are all isometric.

The sharp estimate for the Hausdorff dimension of the singular set of local perimeter minimizers in noncollapsed RCD(K,n) spaces shown in [44] states the following: if $(X,d,\mathcal {H}^n)$ is an RCD(K,n) space without boundary and $E \subset X$ is a locally perimeter minimizing set, then

$$\begin{aligned} \textrm{dim}_\mathcal {H}(\mathcal {S}^E) \le n-3. \end{aligned}$$

The Hausdorff dimension of the singular set of a noncollapsed RCD(K,n) space without boundary is at most $n-2$, as shown in [43]. The analogous result for noncollapsed Ricci limit spaces was shown in [24]. In the case of noncollapsed limits of manifolds with two-sided bounds on the Ricci curvature, a stronger estimate on the Hausdorff dimension of the singular set holds. It was shown in [28] that if (*X*, *d*, *x*) is a noncollapsed limit of manifolds with two-sided bounds on the Ricci curvature of dimension *n*, then the Hausdorff dimension of its singular set is at most $n-4$.

Comparing the estimates for $\textrm{dim}_\mathcal {H}(\mathcal {S}^E)$ and $\textrm{dim}_\mathcal {H}(\mathcal {S}(X))$ when $E \subset X$ is a locally perimeter minimizer in a noncollapsed RCD(K,n) space, a question that arises is whether, under the stronger assumption that *X* is a noncollapsed limit of manifolds with two-sided bounds on the Ricci curvature, it holds $\textrm{dim}_\mathcal {H}(\mathcal {S}^E) \le n-5$. This is the content of the main result of this note.

### Theorem 1

Let (*X*, *d*, *p*) be a noncollapsed limit of manifolds with two-sided bounds on the Ricci curvature of dimension *n*. If $E \subset X$ is a locally perimeter minimizing set, then $\mathcal {S}^E=\mathcal {S}^E_{n-5}$. In particular, it holds

1 $$\begin{aligned} \textrm{dim}_{\mathcal {H}}(\mathcal {S}^E) \le n-5. \end{aligned}$$

Let us point out that Theorem 1 is sharp, as shown in Example 3.11.

### Remark 1.1

(On the behaviour of $\partial E$ in $\mathcal {R}(X)$) Let $E \subset X$ be as in Theorem 1. By [44], the Hausdorff dimension of $\mathcal {S}^E \cap \mathcal {R}(X)$ is at most $n-8$. Exploiting the presence of the double bound on the Ricci curvature, one obtains a finer description of $\partial E \cap \mathcal {R}(X)$. By [11, 23], the metric space *X* is a $C^{1,\alpha }$ manifold for every $\alpha \in (0,1)$ outside of its singular set. By means of classical regularity results, one can then show that $\partial E \cap \mathcal {R}(X)$ is a $C^{1,\alpha }$ hypersurface of $\mathcal {R}(X)$ for every $\alpha \in (0,1)$ outside of a closed set of Hausdorff dimension at most $n-8$ (see Proposition 3.12).

Before outlining the proof of Theorem 1, we mention that the key step in the proof is Theorem 2, which is a Bernstein-type theorem for cones (see the comment before Lemma 2.20 for a definition of a cone) over manifolds of constant curvature 1.

### Theorem 2

Let (*M*, *g*) be a manifold of constant sectional curvature equal to 1 and of dimension $n \le 6$. Let *C*(*M*) be the metric cone over *M* and let *p* be its tip. If $E \subset C(M)$ is a perimeter minimizing set such that $p \in \partial E$, then $M \cong S^n$, $C(M) \cong \mathbb {R}^{n+1}$ and $E \subset C(M) \cong \mathbb {R}^{n+1}$ is a half space.

The proof of the previous theorem follows by adapting a classical result of Simons from [45]. Let us mention that in Theorem 2 the assumption on the dimension is sharp, as shown by Example 3.10.

We now outline the proof of Theorem 1. The estimate on the Hausdorff dimension (1) follows from the stratification result [37, Theorem 4.1] and $\mathcal {S}^E = \mathcal {S}^E_{n-5}$.

We divide the proof of $\mathcal {S}^E = \mathcal {S}^E_{n-5}$ in two steps. We first show that $\mathcal {S}^E = \mathcal {S}^E_{n-4}$. We rely on the following argument: suppose by contradiction that $x \in \mathcal {S}^E {\setminus } \mathcal {S}^E_{n-4}$. Then, by [28, Theorem 5.12], the tangent space to *X* at *x* is isometric to $\mathbb {R}^n$. Moreover, by assumption, a tangent space to *E* at *x* is of the form $\mathbb {R}^{n-3} \times A$, where $A \subset \mathbb {R}^3$ is itself a perimeter minimizer. Therefore, by a classical Bernstein-type theorem (see, for instance, [41, Theorem 28.17]), it follows that *A* is a half space. This provides the desired contradiction and shows $\mathcal {S}^E = \mathcal {S}^E_{n-4}$.

In a second step of the proof we show $\mathcal {S}^E_{n-4} {\setminus } \mathcal {S}^E_{n-5} = \emptyset$. To this end, we suppose by contradiction that $x \in \mathcal {S}^E_{n-4} {\setminus } \mathcal {S}^E_{n-5}$. Then, by [28] and [11] (see also [27, Theorem 1.16]), a tangent space of *X* at *x* is $\mathbb {R}^{n-4} \times C(S^3/\Gamma )$, where $\Gamma \subset O(4)$ is a discrete group of isometries of the sphere acting freely.

Moreover, a tangent space to *E* at *x* is of the form $\mathbb {R}^{n-4} \times A$, where $A \subset C(S^3/\Gamma )$ is itself a perimeter minimizer.

Therefore, by Theorem 2, we are able to conclude $\Gamma = \{id_{S^3}\}$, $C(S^3/\Gamma ) \cong \mathbb {R}^4$, and $A \cong \mathbb {R}^3 \times [0,+\infty )$, contradicting the initial assumption $x \in \mathcal {S}^E_{n-4} \setminus \mathcal {S}^E_{n-5}$.

## Preliminaries

Throughout the paper, a *pointed metric space* is a triple (*X*, *d*, *x*), where (*X*, *d*) is a complete and separable metric space and $x \in X$. We write $B_r(x)$ for the open ball centered at $x\in X$ of radius $r>0$. Under our working conditions, the closed metric balls are compact, so we assume from the beginning the metric space (*X*, *d*) to be proper. We denote by $n \ge 0$ the Hausdorff dimension of (*X*, *d*), and by $\mathcal {H}^n$ the corresponding Hausdorff measure.

Given a positive Borel measure $\mathfrak {m}$ on *X*, we denote by $L^0(X)$ the set of real valued measurable functions. For every $p>0$, we denote by

$$\begin{aligned} L^p(X):= \left\{ u \in L^0(X): \int _X |u|^p \, d \mathfrak {m}< \infty \right\} \end{aligned}$$

the space of *p*-integrable functions. Given a function , we define its local Lipschitz constant at $x \in X$ byand $\textrm{lip}(u)(x) = 0$ otherwise. We indicate by $\textrm{LIP}(X)$ and $\textrm{LIP}_\textrm{loc}(X)$ the space of Lipschitz functions, and locally Lipschitz functions respectively. Moreover, given a set $E \subset X$, we denote by $\chi _E$ its characteristic function.

When we refer to pointed Riemannian manifolds (*M*, *d*, *x*), we implicitly mean that *M* is a smooth manifold equipped with a Riemannian metric which induces the Riemannian distance *d*.

### Ricci Limit Spaces

The starting point of the theory of Ricci limit spaces is the notion of Gromov-Hausdorff convergence. For an overview of the topic see [21, 47], for instance.

#### Definition 2.1

(Pointed Gromov-Hausdorff convergence) A sequence of pointed metric spaces $(X_k, d_k, x_k)$ is said to converge in the pointed Gromov-Hausdorff topology to (*X*, *d*, *x*) if there exists a separable metric space $(Z,d_Z)$ and isometric embeddings $i_k: X_k \xrightarrow [] Z$ and $i: X \xrightarrow [] Z$ such that, for any $\varepsilon >0$ and $r>0$ there exists $\bar{k} \in \mathbb {N}$ such that for for every $k \ge \bar{k}$ we have

$$\begin{aligned} i\left( B_r^X(x)\right) \subset B_\varepsilon ^Z\left( i_k\left( B_{r+\varepsilon }^{X_k}(x_k)\right) \right) , \end{aligned}$$

and

$$\begin{aligned} i_k\left( B_r^{X_k}(x_k)\right) \subset B_\varepsilon ^Z\Big (i_k\Big (B_{r+\varepsilon }^{X}(x)\Big ) \Big ). \end{aligned}$$

We denote the pointed Gromov-Hausdorff convergence by $X_k {\mathop {\longrightarrow }\limits ^{p\textrm{GH}}} X$ and we say that *Z* is the space realizing the convergence.

Let us recall the definition of the spaces we work with.

#### Definition 2.2

(Noncollapsed limits of manifolds with two-sided bounds on the Ricci curvature ) Let $(M_k,d_k,x_k)$ be a sequence of pointed Riemannian manifolds of fixed dimension $n \ge 2$ such that

2 $$\begin{aligned} \textrm{Ric}_{M_k} \ge -(n - 1). \end{aligned}$$

Suppose there exists a metric space (*X*, *d*, *x*) such that $M_k {\mathop {\longrightarrow }\limits ^{p\textrm{GH}}} X$. Then we say that *X* is a Ricci limit space. If condition (2) is strengthened to

$$\begin{aligned} |\textrm{Ric}_{M_k}| \le n - 1, \end{aligned}$$

we say that *X* is a limit of manifolds with two-sided bounds on the Ricci curvature. Moreover, if

3 $$\begin{aligned} \textrm{vol}(B_1(x_k)) \ge v > 0 \end{aligned}$$

holds, we say that *X* is noncollapsed.

Ricci limit spaces were studied extensively in [24, 25] and [26]. In particular, it was shown that the non-collapsing assumption (3) forces the Hausdorff dimension of the limit space and that of the approximating sequence to be the same.

Let us recall the definition of tangent space in the setting of metric spaces.

#### Definition 2.3

(Tangent space to a metric space at a point) Let (*X*, *d*) be a metric space and let $x \in X$. We define the *space of tangent spaces* at *x*, denoted by $\textrm{Tan}(X,x)$, to be the set of all $(Y,d_Y,y)$ such that there exists a sequence $1> r_k > 0$, $r_k \searrow 0$ that satisfies

$$\begin{aligned} (X, d / r_k, x) {\mathop {\longrightarrow }\limits ^{p\textrm{GH}}} (Y, d_Y, y). \end{aligned}$$

In the case of Ricci limit spaces, Gromov compactness Theorem implies that the set of tangent spaces is always non-empty. Moreover, in [24] it was shown that for noncollapsed Ricci limit spaces all tangent spaces are metric cones (see [21] for the definition of metric cones).

We now report the notion of singular stratum of a Ricci limit space.

#### Definition 2.4

(Singular Stratum of a Ricci limit space) Let *X* be a noncollapsed Ricci limit space. For every $k \in \mathbb {N}$ the *k*-singular stratum is defined to be

$$\begin{aligned} \mathcal {S}_{k}:= \left\{ x \in X: \ \text{ no } \text{ tangent } \text{ space } \text{ is } \text{ isometric } \text{ to } \mathbb {R}^{k+1} \times Y \text{ for } \text{ some } \text{ metric } \text{ space } Y\right\} . \end{aligned}$$

It was proved in [24] that the Hausdorff dimension of $\mathcal {S}^{k}$ is always less or equal to *k*.

The structure of singular strata of noncollapsed limits of manifolds with two-sided bounds on the Ricci curvature was studied in [28]. A key result used in the proof of Theorem 1 is Theorem 2.5 below, which is an immediate consequence of [28, Theorem 5.12]. In the next statement, if $k \in \mathbb {Z} \setminus \mathbb {N}$, we set $\mathcal {S}_k:=\emptyset$.

#### Theorem 2.5

Let (*X*, *d*, *x*) be a noncollapsed limit of manifolds with two-sided bounds on the Ricci curvature of dimension $n \ge 2$. Then $\mathcal {S}_{n-1} \setminus \mathcal {S}_{n-4}= \emptyset$.

The following two results follow from [11, 23].

#### Theorem 2.6

For every $n \in \mathbb {N}$, $n \ge 2$ and $\delta ,\alpha \in (0,1)$, there exists $\epsilon >0$ satisfying the following. Let $(M^n,g,p)$ be a pointed manifold with $|\textrm{Ric}_{M_k}| \le \epsilon ,$ and such that $B_2(p)$ is $\epsilon$-close in the GH distance to the Euclidean ball $B_2(0^n)$. Then, there exists harmonic coordinates in *M* around *p* such that

$$\begin{aligned} \Vert g_{ij}-\delta _{ij}\Vert _{C^{1,\alpha }(B_1(0^n))} \le \delta . \end{aligned}$$

#### Theorem 2.7

Let (*X*, *d*, *x*) be a noncollapsed limit of manifolds with two-sided bounds on the Ricci curvature of dimension $n \ge 2$. For every $\alpha \in (0,1)$, *X* is an open $C^{1,\alpha }$ Riemannian manifold outside of $S_{n-4}$.

Another key result used in the proof of Theorem 1 is Theorem 2.8 below. Given a metric space (*X*, *d*), the *metric cone over X* is defined as the set

$$\begin{aligned} C(X):=(X \times [0,+\infty ))/{\sim } \quad \text {with } (x,0) \sim (y,0) \text { for every } x,y \in X, \end{aligned}$$

equipped with the cone metric (see [21] for the definition). The point $(x, 0) \in C(X)$ is called the tip of *C*(*X*).

#### Theorem 2.8

Let (*X*, *d*, *x*) be a noncollapsed limit of manifolds with two-sided bounds on the Ricci curvature. Then, for any $x \in \mathcal {S}_{n-4} {\setminus } \mathcal {S}_{n-5}$, there exists a tangent space at *x* which is isometric to $\mathbb {R}^{n-4} \times C(S^3/\Gamma )$, where $\Gamma \subset O(4)$ is a discrete group acting freely.

#### Proof

The proof follows adapting the one from [27, Theorem 1.16]. We report the argument for the sake of completeness. Assume by contradiction that $x \in \mathcal {S}_{n-4} {\setminus } \mathcal {S}_{n-5}$. Then, there exists a tangent space to *X* at *x* of the form $\mathbb {R}^{n-4} \times C(Y)$, for some metric space *Y*. By [28] and [11], it follows that *Y* is a three dimensional smooth Riemannian manifold and that the Ricci curvature of *C*(*Y*) outside of the tip vanishes. This implies that *Y* has constant sectional curvature equal to 1, so that it is a quotient $S^3/\Gamma$, where $\Gamma \subset O(4)$ is a discrete group acting freely. $\square$

### Finite Perimeter Sets and Perimeter Minimizers

A *metric measure space* is a triple $(X,d,\mathfrak {m})$, where (*X*, *d*) is a complete and separable metric space and $\mathfrak {m}$ is a non negative Borel measure on *X*, which is finite on metric balls. Given $x \in X$, quadruples of the form $(X,d,\mathfrak {m},x)$ are called *pointed metric measure spaces*. We say that two pointed metric measure spaces $(X,d,\mathfrak {m},x)$ and $(Y,\rho , \mu ,y)$ are isometric in the sense of pointed metric measure spaces if there exists an isometry in the sense of metric spaces $i: X \xrightarrow [] Y$ such that $i_{\#}\mathfrak {m}= \mu$ and $i(x) = y$. In the case of noncollapsed Ricci limit spaces, the reference measure $\mathfrak {m}$ corresponds to the Hausdorff measure $\mathcal {H}^n$.

For the purposes of this work, we need to introduce some background regarding finite perimeter sets and perimeter minimizers in Ricci limit spaces. In recent years, this theory has been developed in the more general framework of metric measure spaces with a synthetic notion of lower Ricci curvature bounds (RCD), which includes Ricci limit spaces. For an account of the theory of RCD spaces we refer to the survey [3].

In this section, we restrict our attention to noncollapsed RCD(K,n) spaces, that is RCD(K,n) spaces where the reference measure is the Hausdorff measure $\mathcal {H}^n$ (see [32]). The reason for this choice is that some of the results we report here were obtained in such setting. Let us remark that the family of noncollapsed Ricci limit spaces is a subset of the class of noncollapsed RCD(K,n) spaces.

The theory of finite perimeter sets in RCD spaces was developed in [6, 13, 17, 19, 20, 44], among others.

We begin by recalling the definition of finite perimeter sets in the setting of metric measure spaces (see [4, 7, 42]).

#### Definition 2.9

(Sets of locally finite perimeter) Let $(X,d,\mathfrak {m})$ be a metric measure space and let $E \subset X$ be a Borel set. Given an open set $A \subset X$, the perimeter of *E* in *A* is defined to be

$$\begin{aligned} P(E, A):= \inf \left\{ \liminf _{k \xrightarrow [] \infty } \int _A \textrm{lip} f_k\, d\mathfrak {m}\, \ f_k \in \textrm{LIP}_\textrm{loc}(A), \ f_k \xrightarrow [] \chi _E\ \text{ in } L^1_\textrm{loc}(A)\right\} . \end{aligned}$$

The set $E \subset X$ is said to have locally finite perimeter if $P(E, B_r(x)) < + \infty$ for all $x \in X$ and $r > 0$.

Let us point out that, if *E* has locally finite perimeter, there exists a unique Radon measure $\mu$ such that $\mu (A)=P(E,A)$ if $A \subset X$ is open. This measure is denoted $P(E,\cdot )$.

The following lemma, which follows immediately from Definition 2.9, is used in the proof of Theorem 2.

#### Lemma 2.10

Consider two metric spaces $(X,d_x)$ and $(Y,d_y)$ with a bijective isometry $f:X \xrightarrow [] Y$. If both spaces are equipped with Hausdorff measures of the same dimension, then for every $A,B \subset X$ we have

$$\begin{aligned} P(A,B)=P(f(A),f(B)). \end{aligned}$$

In order to recall the notion of $L^1$ convergence of sets along a converging sequence of spaces introduced in [10] and [6], we first need to report the definition pointed measured Gromov-Hausdorff convergence, which extends Definition 2.1.

#### Definition 2.11

(Pointed measured Gromov-Hausdorff convergence) A sequence of pointed metric measure spaces $(X_k, d_k, \mathfrak {m}_k, x_k)$ is said to converge in the pointed measured Gromov-Hausdorff topology to $(X,d,\mathfrak {m},x)$ if $X_k {\mathop {\longrightarrow }\limits ^{p\textrm{GH}}} X$ and, using the same notation of Definition 2.1, we have that $({i_k})_\# \mathfrak {m}_k \rightharpoonup i_\# \mathfrak {m}$ w.r.t. continuous functions with bounded support on *Z*. We denote the pointed measured Gromov-Hausdorff convergence by $X_k {\mathop {\longrightarrow }\limits ^{pm\textrm{GH}}} X$ and we say that *Z* is the space realizing the convergence.

In the case of pointed Ricci limit spaces $(X_k,d_k,x_k)$ of the same dimension $n\ge 2$ satisfying

$$\begin{aligned} \mathcal {H}_k^n(B_1(x_k))> v > 0, \end{aligned}$$

where $\mathcal {H}^n_k$ is the Hausdorff measure relative to $d_k$, the pointed Gromov-Hausdorff convergence (Definition 2.1) to a metric space (*X*, *d*, *x*) is equivalent to the pointed measured Gromov-Hausdorff convergence (Definition 2.11) of $(X_k,d_k, \mathcal {H}_k^n, x_k)$ to $(X, d, \mathcal {H}^n, x)$. For a proof of this fact, see [32, Theorem 1.2].

Let us report the definition of $L^1$ convergence of sets.

#### Definition 2.12

($L^1$ convergence of sets) Let $(X_k,d_k,\mathfrak {m}_k,x_k)$ be a sequence of pointed metric measure spaces converging in pointed measured Gromov-Hausdorff sense to $(X,d,\mathfrak {m},x)$. We say that a sequence of Borel sets  with $\mathfrak {m}_k(E_k) < \infty$ converges in $L^1$ sense to $E \subset X$ if, given an embedding space $(Z,d_Z)$ as in Definition 2.11, we have $\chi _{E_k}\mathfrak {m}_k \rightharpoonup \chi _{E}\mathfrak {m}$ in duality with continuous boundedly supported functions in *Z* and . Moreover, $E_k$ is said to converge in $L^1_\textrm{loc}$ to *E* if the sets $E_k \cap B_r(x_k)$ converge in $L^1$ to $E \cap B_r(x)$ for all $r>0$.

Let us point out that, in the case where $(X_k,d_k, \mathfrak {m}_k, x_k)$ is isometric to $(X, d, \mathfrak {m}, x)$ in the sense of pointed metric measure spaces for all *k*, $L^1$ ($L^1_\textrm{loc}$) convergence in the sense of Definition 2.12 is equivalent to requiring that $\mathfrak {m}(E \Delta E_k) \xrightarrow [] 0$ ($\mathfrak {m}((E \Delta E_k)\cap B_r(x)) \xrightarrow [] 0$ for all $r > 0$).

We report the definition of tangent space to a finite perimeter set found in [6]. The set of tangent spaces to a noncollapsed RCD(K,n) space is defined analogously to Definition 2.3, where the pointed Gromov-Hausdorff convergence is replaced by convergence in the pointed measured Gromov-Hausdorff sense.

Given a noncollapsed RCD(K,n) space $(X,d,\mathcal {H}^n)$ and $x \in X$, we still refer to the set of tangent spaces at *x* as Tan(*X*, *x*). The regular set of *X* is defined as $\mathcal {R}(X):= \{x \in X: \textrm{Tan}(X,x) = \{(\mathbb {R}^n, d_\textrm{eucl}, 0) \}\}$. The singular set is defined as $\mathcal {S}(X):=X \setminus \mathcal {R}(X)$. By a result shown in [32], it holds

$$\begin{aligned} \mathcal {R}(X) = \{x \in X:(\mathbb {R}^n,d_{\textrm{eucl}}, 0) \in \textrm{Tan}(X,x)\}\,. \end{aligned}$$

#### Definition 2.13

(Tangents to a set of locally finite perimeter) Let $(X,d,\mathcal {H}^n)$ be a noncollapsed $\textrm{RCD}$(K,n) space and let $E\subset X$ be a set of locally finite perimeter. We say that $(Y, d_Y, F, y)\in \textrm{Tan}(X,E,x)$ if the pointed metric measure space $(Y,d_Y,\mathcal {H}^n,y)$ belongs to $\textrm{Tan}(X,x)$ and $F\subset Y$ is a set of locally finite perimeter of positive measure such that $\chi _E$ converges in the $L^1_\textrm{loc}$ sense of Definition 2.12 to *F* along the blow-up sequence associated to the tangent *Y*.

#### Definition 2.14

(Perimeter minimizers) Let $(X,d,\mathfrak {m})$ be a metric measure space and let $E \subset X$ be a set of locally finite perimeter. We say that *E* is locally perimeter minimizing if for every $x \in X$ there exists $r>0$ with the following property: for every $F \subset X$ such that $F \Delta E \subset \subset B_r(x)$ we have $P(E,B_r(x) ) \le P(F,B_r(x))$. Similarly, the set $E \subset X$ is perimeter minimizing if for every $x \in X$, $r>0$ and $F \subset X$ such that $F \Delta E \subset \subset B_r(x)$ we have that $P(E,B_r(x) ) \le P(F,B_r(x) )$.

Let $(X,d,\mathcal {H}^n)$ be a noncollapsed RCD(K,n) space and suppose that $E \subset X$ is a locally perimeter minimizing set. If $(Y,d, F, y) \in \textrm{Tan}(X, E,x)$, then *F* is a perimeter minimizer. For a proof of this fact, we refer to [6, Proposition 3.9]. We report a result regarding density estimates for perimeter minimizing sets in the RCD setting which follows from [40, Theorem 4.2].

#### Lemma 2.15

Let $(X,d,\mathcal {H}^n)$ be an $\textrm{RCD}(0,n)$ space and let $E \subset X$ be a perimeter minimizing set. Then, up to modifying *E* on a $\mathcal {H}^n$-negligible set, there exists $C = C(n) >0$ such that for any $x \in \partial E$ and $r > 0$

$$\begin{aligned} \frac{\mathcal {H}^n(E \cap B_r(x))}{\mathcal {H}^n(B_r(x))} \ge C, \qquad \frac{\mathcal {H}^n(B_r(x)\setminus E)}{\mathcal {H}^n(B_r(x))} \ge C. \end{aligned}$$

Moreover, it follows from [40, Theorem 4.2] that if a set is perimeter minimizing in an RCD(K,n) space, then it admits both an open and a closed representative. These representatives have the same topological boundary. Whenever we refer to the boundary of a locally perimeter minimizing set, we mean the topological boundary of its open (or closed) representative.

#### Definition 2.16

(Regular points of locally perimeter minimizing sets) Let $(X, d,\mathcal {H}^n)$ be a noncollapsed RCD(K,n) space and let $E\subset X$ be a locally perimeter minimizing set in the sense of Definition 2.14. Given $x\in \partial E$, we say that *x* is a regular point of *E* if

$$\begin{aligned} \textrm{Tan}(X,E,x)=\{(\mathbb {R}^n,d_{\textrm{eucl}},\mathbb {R}^{n-1} \times [0,+\infty ),0)\}\,. \end{aligned}$$

The set of regular points of *E* is denoted by $\mathcal {R}^E$. The set of singular points is defined to be $\mathcal {S}^E:= X{\setminus } \mathcal {R}^E$.

By the $\varepsilon$-regularity result shown in [44, Theorem 6.8], it holds

$$\begin{aligned} \mathcal {R}^E = \{x \in \partial E:(\mathbb {R}^n,d_{\textrm{eucl}},\mathbb {R}^{n-1} \times [0,+\infty ),0) \in \textrm{Tan}(X,E,x)\}\,. \end{aligned}$$

We remark that there is a subtle distinction between the notion of regular point for a set of finite perimeter in an RCD space and for a set of finite perimeter in Euclidean space or in a Riemannian manifold. Namely, if one uses the non-smooth definition in the smooth setting, the usual uniqueness requirement for the tangent space needs to be dropped, as different half-spaces are all identified.

We here recall the notion of singular stratum of a perimeter minimizing set.

#### Definition 2.17

(Singular Strata) Let $(X, d,\mathcal {H}^n)$ be an RCD(K,n) space, $E\subset X$ a locally perimeter minimizing set in the sense of Definition 2.14 and $0 \le k \le n-3$ an integer. The *k*-singular stratum of *E*, $\mathcal {S}^E_k$, is defined as

$$\begin{aligned} \begin{aligned} \mathcal {S}_k^E:=&\{x \in \partial E: \text{ no } \text{ element } \text{ of } \textrm{Tan}(X,E,x) \text{ is } \text{ of } \text{ the } \text{ form } (Y,d_Y, F,y), \\&\; \text{ with } (Y,d_Y,y) \text{ isometric } \text{ to } (Z\times \mathbb {R}^{k+1},d_Z \times d_\textrm{eucl},(z,0))\\&\text{ for } \text{ some } \text{ pointed } (Z,d_Z,z)\\&\; \text{ and } F=G\times \mathbb {R}^{k+1} \text{ with } G\subset Z \text{ perimeter } \text{ minimizer }\}. \end{aligned} \end{aligned}$$

A key result that we use in the proof of Theorem 1 is the stratification of the singular set of locally perimeter minimizing sets, which can be found in [37, Theorem 4.5]. This result concerns noncollapsed RCD(K,n) spaces with empty boundary, i.e. such that $\mathcal {S}_{n-1} \setminus \mathcal {S}_{n-2}=\emptyset$ (for more on boundaries of RCD(K,n) spaces see, for instance, [18]). Since Ricci limit spaces have empty boundary (as shown in [24]), the result also applies to our setting.

#### Theorem 2.18

(Stratification of the singular set) Let $(X,d,\mathcal {H}^n)$ be a noncollapsed $\textrm{RCD}(K,n)$ space with empty boundary, and let $E \subset X$ be a locally perimeter minimizing set. Then

$$\begin{aligned} \textrm{dim}_\mathcal {H} \mathcal {S}_k^E \le k \quad \text{ for } k = 0,1,..., n-3. \end{aligned}$$

Moreover, $$\mathcal {S}^E\setminus \mathcal {S}_{n-2}^E = \emptyset $$.

Let us recall a technical lemma. A proof of the equivalent statement in Euclidean spaces can be found in [41, Lemma 28.13], whereas a proof of Lemma 2.19 can be found in [37, End of Step 3 in the proof of Theorem 4.4].

#### Lemma 2.19

Let $(X, d, \mathcal {H}^n)$ be a noncollapsed $\textrm{RCD}(K,n)$ space. Let $k \in \mathbb {N}$ and let $A \times \mathbb {R}^k \subset X \times \mathbb {R}^k$ be a perimeter minimizing set, then $A \subset X$ is also perimeter minimizing.

We conclude the section by reporting a useful result regarding tangent spaces to perimeter minimizing sets in cones. Given a metric cone *C*(*X*) and a subset $A \subset X$, the cone *C*(*A*) can be canonically identified with a subset of *C*(*X*), and this identification is implicitly used in the statement of the next lemma.

#### Lemma 2.20

Let (*C*(*X*), *d*) be a metric cone such that $(C(X),d,\mathcal {H}^n)$ is a noncollapsed $\textrm{RCD}(0,n)$ space. If $E \subset C(X)$ is a perimeter minimizing set whose boundary contains the tip of the cone *p*, then there exists a set $A \subset X$ such that $C(A) \subset C(X)$ is a perimeter minimizing cone, whose boundary contains *p*, and such that

$$\begin{aligned} (C(X), d, C(A), p) \in \textrm{Tan}(C(X), E, p). \end{aligned}$$

The proof is a consequence of the rigidity part of the Monotonicity Formula found in [37, Theorem 3.1] and a density estimate for locally perimeter minimizing sets shown in [40, Lemma 5.1].

### Second Variation Formula in Euclidean Spaces

In this section we collect some technical results on perimeter minimizing sets in Euclidean spaces that are used in the proof of Theorem 2. The topic is classical and we refer to [41] and [39] for an account of the theory.

We now recall the notion of tangential derivatives to the boundary of a smooth open set $E \subset \mathbb {R}^n$. Let $\nu : \partial E \xrightarrow [] S^{n-1}$ be the outward normal vector on $\partial E$ and let $g \in C^{\infty }(\mathbb {R}^n)$. On a point $x \in \partial E$ the tangential derivative of *g* is defined as

$$\begin{aligned} \nabla _E g:=\nabla g - \nu (\nabla g \cdot \nu ). \end{aligned}$$

Given any integer $1 \le i \le n$, the *i*-th component of the tangential derivative of *g* is defined as

$$\begin{aligned} \nabla _{E,i} g:=\partial _i g -\nu _i(\nabla g \cdot \nu ). \end{aligned}$$

Similarly, the tangential Laplacian of *g* is defined as

$$\begin{aligned} \Delta _Eg:=\sum _{i=1}^n \nabla _{E,i} \nabla _{E,i} g. \end{aligned}$$

Both $\nabla _E g$ and $\Delta _E g$ depend only on the restriction of *g* to $\partial E$; for this reason we consider tangential derivatives and tangential Laplacians of functions that are defined only on $\partial E$, assuming implicitly that we are extending the functions smoothly to $\mathbb {R}^n$ before applying such operators.

We denote by  the norm of the second fundamental form of $\partial E$ in $\mathbb {R}^n$. This coincides with the square root of the sum of the squares of the principal curvatures of $\partial E$. One can check that $|\Pi _E|^2=\sum _{i,j}(\nabla _{E,i} \nu _j)^2$ (see [39, Remark 10.6]). Whenever $\partial E$ is not smooth, we assume that the aforementioned objects are defined in the largest smooth subset of $\partial E$. We mention that in [39] the objects $\nabla _E$, $\nabla _{E,i}$, $\Delta _E$, $|\Pi _E|$ are denoted respectively by $\delta , \delta _i, \mathcal {D}$, and *c*.

We say that a set  is a cone with tip *p* if it is invariant under dilations which fix *p*. This notion is consistent with the one of metric cone previously introduced. Without loss of generality, in the remainder of this work we suppose that *p* coincides with the origin $0 \in \mathbb {R}^n$.

#### Remark 2.21

By inspecting the proof of [39, Lemma 10.9] one realizes that if $E \subset \mathbb {R}^n$ is a cone which is both smooth and has zero mean curvature in , then $|\Pi _E|^2$ is homogeneous of degree $-2$.

We now recall the second variation formula for sets with vanishing mean curvature, which can be found in [39, Identity (10.13)].

#### Proposition 2.22

Let $E \subset \mathbb {R}^n$ be an open set such that $\partial E$ is smooth and has zero mean curvature in an open set $A \subset \subset \mathbb {R}^n$. Let $\nu :A \xrightarrow [] S^{n-1}$ be an extension of the outward unit normal of $\partial E$ to *A*, and let $\zeta \in C^{\infty }_c(A)$. Define  by $F_t(x):=x+t\zeta (x) \nu (x)$ and set $E_t:=F_t(E)$. Then

$$\begin{aligned} \Big ( \frac{d^2}{dt^2}P(A,E_t)\Big )_{|t=0}= \int _{\partial E}(|\nabla _E \zeta |^2-|\Pi _E|^2\zeta ^2) \, d\mathcal {H}^{n-1}. \end{aligned}$$

We conclude the section by recalling that tangential derivatives satisfy an integration by parts formula and that $\Delta _E |\Pi _E|^2$ is well behaved on minimal sets that are invariant under dilations. The next result can be found in [39, Lemma 10.8].

#### Lemma 2.23

Let $E \subset \mathbb {R}^n$ be such that $\partial E$ is a smooth hypersurface and let $\phi \in C^{\infty }_c(\mathbb {R}^n)$. Then

$$\begin{aligned} \int _{\partial E} \nabla _{E,i} \phi \, d\mathcal {H}^{n-1}=-\int _{\partial E} \phi \nu _i \, d\mathcal {H}^{n-1}. \end{aligned}$$

The next result can be found in [39, Lemma 10.9].

#### Lemma 2.24

Let $E \subset \mathbb {R}^n$ be a cone which is both smooth and has vanishing mean curvature in $\mathbb {R}^n \setminus \{0\}$. Then in $\partial E \cap \mathbb {R}^n \setminus \{0\}$ we have

$$\begin{aligned} \frac{1}{2} \Delta _E |\Pi _E|^2 \ge -|\Pi _E|^4+|\nabla _E |\Pi _E||^2+ \frac{2|\Pi _E|^2}{|x|^2}. \end{aligned}$$

## Proof of the Main Results

The goal of this section is to prove Theorem 1 and Theorem 2, which we restate for the reader’s convenience. The proofs will be given at the end of the section.

### Theorem 1

Let (*X*, *d*, *p*) be a noncollapsed limit of manifolds with two-sided bounds on the Ricci curvature of dimension *n*. If $E \subset X$ is a locally perimeter minimizing set, then $\mathcal {S}^E=\mathcal {S}^E_{n-5}$. In particular, it holds

$$\begin{aligned} \textrm{dim}_{\mathcal {H}}(\mathcal {S}^E) \le n-5. \end{aligned}$$

### Theorem 2

Let (*M*, *g*) be a manifold of constant sectional curvature equal to 1 and of dimension $n \le 6$. Let *C*(*M*) be the metric cone over *M* and let *p* be its tip. If $E \subset C(M)$ is a perimeter minimizing set such that $p \in \partial E$, then $M \cong S^n$, $C(M) \cong \mathbb {R}^{n+1}$ and $E \subset C(M) \cong \mathbb {R}^{n+1}$ is a half space.

Let us fix some notation. In this section $n \in \mathbb {N}$, $n \ge 2$ and $\Gamma$ is a discrete group of isometries of $S^{n-1}$ acting freely. Moreover, $\Gamma$ induces an action on $\mathbb {R}^n$ given in polar coordinates by $g \cdot (\omega , r):=(g(\omega ), r)$. We denote by  the projection on the quotient space. Since $\Gamma$ acts freely on $S^{n-1}$, it follows that it also acts freely on $\mathbb {R}^n\setminus \{0\}$. Consequently, $\pi _{|\mathbb {R}^n\setminus \{0\}}$ is a covering of $(\mathbb {R}^n{\setminus } \{0\}) /\Gamma$. Therefore, it is also a local isometry.

We say that an open set $U \subset ({\mathbb {R}^n\setminus \{0\}}) / \Gamma$ is a *cover chart* if its preimage through $\pi$ is a finite union of disjoint open sets $\{U_i\}_{i =1}^l$ (where *l* is the cardinality of $\Gamma$) such that $\pi _{|U_i}:U_i \xrightarrow [] U$ is a bijective isometry for every *i*. Given a subset $E \subset \mathbb {R}^n$ and $g \in \Gamma$, we denote $g \cdot E:=\{g \cdot e: e \in E\}$. Moreover, given a subset $E \subset \mathbb {R}^n$ ($\mathbb {R}^n/\Gamma$) and $t > 0$, we define the rescaled set $E/t:= \{x \in \mathbb {R}^n (\mathbb {R}^n/\Gamma ): t x \in E\}$.

### Definition 3.1

($\Gamma$-symmetric sets) We say that a set $E \subset \mathbb {R}^n$ is $\Gamma$-*symmetric* if for every $g \in \Gamma$ we have $g \cdot E=E$.

The next lemma shows that $\Gamma$-symmetric sets arise as preimages via $\pi$ of sets in $\mathbb {R}^n/ \Gamma$.

### Lemma 3.2

If $E \subset \mathbb {R}^n$ is $\Gamma$-symmetric, then $\pi ^{-1}(\pi (E))= E$. Conversely, if $F \subset \mathbb {R}^n /\Gamma$, then $\pi ^{-1}(F)$ is $\Gamma$-symmetric and $\pi (\pi ^{-1}(F))= F$.

### Proof

We start by showing that if $E \subset \mathbb {R}^n$ is $\Gamma$-symmetric, then $\pi ^{-1}(\pi (E))= E$.

Observe that $\pi ^{-1}(\pi (E)) \supset E$ trivially, so that we only need to prove the other inclusion. If $x \in \pi ^{-1}(\pi (E))$, then $\pi (x)=\pi (y)$ for some $y \in E$. Therefore, there exists $g \in \Gamma$ such that $g \cdot x=y$, giving that $x \in E$ as this set is $\Gamma$-symmetric.

Let us show that, if $F \subset \mathbb {R}^n /\Gamma$, then $\pi ^{-1}(F)$ is $\Gamma$-symmetric and $\pi (\pi ^{-1}(F))= F$.

Consider $x \in \pi ^{-1}(F)$. We show that for every $g \in \Gamma$ we have $g \cdot x \in \pi ^{-1}(F)$. To this aim, note that $\pi (x)=\pi (g \cdot x)$ so that in particular $g \cdot x \in \pi ^{-1}(x) \subset \pi ^{-1}(F)$. Conversely, let $x \notin \pi ^{-1}(F)$. We show that for every $g \in \Gamma$ it holds $g \cdot x \notin \pi ^{-1}(F)$. Indeed, if $g \cdot x \in \pi ^{-1}(F)$, then $x \in \pi ^{-1}(F)$.

Finally, $\pi (\pi ^{-1}(F))= F$ since $\pi$ is surjective. $\square$

### Definition 3.3

($\Gamma$-symmetric sets minimizing the perimeter against $\Gamma$-symmetric competitors) We say that a $\Gamma$-symmetric set $E \subset \mathbb {R}^n$ minimizes the perimeter against $\Gamma$-symmetric competitors if for every $\Gamma$-symmetric set $A \subset \mathbb {R}^n$ and $r>0$ such that $E \Delta A \subset \subset B_r(0)$ we have

$$\begin{aligned} P(E,B_r(0)) \le P(A,B_r(0)). \end{aligned}$$

The following key lemma allows us to compare the perimeter of subsets of $\mathbb {R}^n/\Gamma$ with the perimeter of their preimage through the projection map $\pi$.

### Lemma 3.4

If $F \subset \mathbb {R}^n/\Gamma$ and $l \in \mathbb {N}$ is the cardinality of $\Gamma$, then for every measurable set $U \subset \mathbb {R}^n/\Gamma$, it holds

$$\begin{aligned} l P(F,U)=P(\pi ^{-1}(F),\pi ^{-1}(U)). \end{aligned}$$

### Proof

We claim that we can find a countable collection of disjoint measurable subsets $\{B_i\}_{i \in \mathbb {N}}$ of $\mathbb {R}^n/\Gamma$ which covers $(\mathbb {R}^n \setminus \{0\}) / \Gamma$ and is such that each set $B_i$ is contained in a cover chart of $(\mathbb {R}^n {\setminus } \{0\}) / \Gamma$. To this aim, let $\{A_i\}_{i \in \mathbb {N}}$ be a covering of $(\mathbb {R}^n \setminus \{0\}) / \Gamma$ with cover charts, which exists as every point has a neighborhood which is a cover chart. To obtain a disjoint cover we define $B_1:=A_1$ and $B_{i+1}:=A_{i+1} {\setminus } \cup _{j=1}^i A_j$.

For every $i \in \mathbb {N}$ the preimage $\pi ^{-1}(A_i)$ coincides with the disjoint union $\cup _{j=1}^l A_i^j$, and for every integer $1 \le j \le l$

$$\begin{aligned} \pi _{|A_i^j}:A_i^j \xrightarrow [] A_i \end{aligned}$$

is a bijective isometry. In particular, by Lemma 2.10

$$\begin{aligned} P(F,B_i \cap U)=P((\pi _{|A_i^j})^{-1}(F),(\pi _{|A_i^j})^{-1}(B_i \cap U)) \quad \text {for every } j = 1,...,l. \end{aligned}$$

Since

$$\begin{aligned} A_i^j \cap (\pi _{|A_i^j})^{-1}(F)= A_i^j \cap \pi ^{-1}(F), \end{aligned}$$

it holds

$$\begin{aligned} P(F,B_i \cap U)=P(\pi ^{-1}(F),(\pi _{|A_i^j})^{-1}(B_i \cap U)) \quad \text {for every } j = 1,...,l. \end{aligned}$$

Summing over *j* we then get that for every $i \in \mathbb {N}$ there holds

$$\begin{aligned} l P(F,B_i \cap U)=P(\pi ^{-1}(F),\pi ^{-1}(U \cap B_i)). \end{aligned}$$

The collection $\{\pi ^{-1}(B_i)\}_{i \in \mathbb {N}}$ is also a disjoint cover of $\mathbb {R}^n \setminus \{0\}$ so that, taking into account that $P(F,\{0\})=P(\pi ^{-1}(F),\{0\})=0$, we obtain

$$\begin{aligned}  &   l P(F,U)= \sum _{i \in \mathbb {N}} l P(F,U \cap B_i)\\  &   = \sum _{i \in \mathbb {N}} P(\pi ^{-1}(F),\pi ^{-1}(B_i \cap U))= P(\pi ^{-1}(F),\pi ^{-1}(U)). \end{aligned}$$

$$\square $$

The next lemma shows that there exists a correspondence between perimeter minimizers in $\mathbb {R}^n/\Gamma$ and $\Gamma$-symmetric sets minimizing the perimeter against $\Gamma$-symmetric competitors in $\mathbb {R}^n$.

### Lemma 3.5

Let $F \subset \mathbb {R}^n/\Gamma$ be a perimeter minimizing set, then $\pi ^{-1}(F)$ is a $\Gamma$-symmetric set minimizing the perimeter against $\Gamma$-symmetric competitors in $\mathbb {R}^n$. Conversely, if $E \subset \mathbb {R}^n$ is a $\Gamma$-symmetric set minimizing the perimeter against $\Gamma$-symmetric competitors, then $\pi (E) \subset \mathbb {R}^n / \Gamma$ is a perimeter minimizing set.

### Proof

Let $F \subset \mathbb {R}^n/\Gamma$ be a perimeter minimizing set. Then $\pi ^{-1}(F)$ is a $\Gamma$-symmetric set by Lemma 3.2. We now show that $\pi ^{-1}(F)$ also minimizes the perimeter with respect to $\Gamma$-symmetric competitors. Let $r > 0$ and $E' \subset \mathbb {R}^n$ be a $\Gamma$-symmetric set such that $\pi ^{-1}(F) \Delta E' \subset \subset B_r(0)$. Since $\pi (B_r(0)) = B_r(0) \subset \mathbb {R}^n / \Gamma$, using Lemma 3.4 we obtain

$$\begin{aligned} P(\pi ^{-1}(F),B_r(0))=l^{-1} P(F,B_r(0)) \le l^{-1} P(\pi (E'),B_r(0))= P(E',B_r(0)). \end{aligned}$$

Since $r > 0$ is arbitrary, we conclude that $\pi ^{-1}(F)$ is a $\Gamma$ symmetric set minimizing the perimeter against $\Gamma$-symmetric competitors in $\mathbb {R}^n$.

In an analogous fashion, one can show that if $E \subset \mathbb {R}^n$ is a $\Gamma$-symmetric set minimizing the perimeter against $\Gamma$-symmetric competitors, then $\pi (E) \subset \mathbb {R}^n / \Gamma$ is a perimeter minimizing set. $\square$

The next proposition shows that $\Gamma$-symmetric sets minimizing the perimeter against $\Gamma$-symmetric competitors in $\mathbb {R}^n$ are locally perimeter minimizing in $\mathbb {R}^n \setminus \{0\}$.

### Proposition 3.6

Let $E \subset \mathbb {R}^n$ be a $\Gamma$-symmetric set minimizing the perimeter against $\Gamma$-symmetric competitors in $\mathbb {R}^n$, then *E* is locally perimeter minimizing in $\mathbb {R}^n \setminus \{0\}$. In particular, in $\mathbb {R}^n \setminus \{0\}$ the set *E* admits an open and a closed representative sharing the same topological boundary. Moreover, if $n \le 7$, then *E* has smooth boundary with vanishing mean curvature in $\mathbb {R}^n \setminus \{0\}$.

### Proof

By Lemma 3.5, the set $\pi (E) \subset \mathbb {R}^n /\Gamma$ is perimeter minimizing. Since the restricted projection map $\pi :\mathbb {R}^n \setminus \{0\} \xrightarrow [] (\mathbb {R}^n/\Gamma ) \setminus \{0\}$ is a local isometry, *E* is then locally perimeter minimizing. $\square$

When we refer to a $\Gamma$-symmetric set minimizing the perimeter against $\Gamma$-symmetric competitors in $\mathbb {R}^n$, we implicitly mean its open representative. In the next lemma we deal with tangent spaces to sets of finite perimeter in $\mathbb {R}^n$ and $\mathbb {R}^n/\Gamma$. In both cases, when referring to elements of the tangent space at a point, we omit the distance.

### Lemma 3.7

Let $E \subset \mathbb {R}^n$ be a $\Gamma$-symmetric set which minimizes the perimeter against $\Gamma$-symmetric competitors and whose boundary contains 0. Then there exists a $\Gamma$-symmetric cone $E' \subset \mathbb {R}^n$ which minimizes the perimeter against $\Gamma$-symmetric competitors, whose boundary contains 0, and such that

$$\begin{aligned} (\mathbb {R}^n,E',0) \in \textrm{Tan}(\mathbb {R}^n, E, 0). \end{aligned}$$

### Proof

By Lemma 3.5, $\pi (E) \subset \mathbb {R}^n/\Gamma$ is a perimeter minimizing set. In particular, by Lemma 2.20, there exists a perimeter minimizing cone $\pi (E)^\infty \subset \mathbb {R}^n /\Gamma$, whose boundary contains 0, and such that

$$\begin{aligned} (\mathbb {R}^n/\Gamma ,\pi (E)^\infty ,0) \in \textrm{Tan}(\mathbb {R}^n/\Gamma ,\pi (E),0). \end{aligned}$$

We claim that

4 $$\begin{aligned} (\mathbb {R}^n,\pi ^{-1}(\pi (E)^\infty ),0) \in \textrm{Tan}(\mathbb {R}^n, E, 0). \end{aligned}$$

We fix $x \in (\mathbb {R}^n \setminus \{0\})/\Gamma$ and we consider a bounded cover chart $A \subset (\mathbb {R}^n \setminus \{0\}) /\Gamma$ containing *x*. The preimage $\pi ^{-1}(A)$ coincides with the disjoint union of open sets $\cup _{j=1}^l A_j$ such that the restricted maps $\pi _{|A_j}:A_j \xrightarrow [] A$ are bijective isometries. Since $(\mathbb {R}^n/\Gamma ,\pi (E)^\infty ,0) \in \textrm{Tan}(\mathbb {R}^n/\Gamma ,\pi (E),0)$, there exists a sequence $t_k \xrightarrow [] 0$, independent of *A*, such that

$$\begin{aligned} \Vert 1_{\pi (E)/t_k}- 1_{\pi (E)^\infty }\Vert _{L^1(A)} \xrightarrow [] 0 \quad \text {as }t_k \xrightarrow [] 0. \end{aligned}$$

Taking into account that $\pi ^{-1}(\pi (E)/t_k)=E/t_k$, for every $j=1,...,l$ it holds

5 $$\begin{aligned} \Vert 1_{E/t_k}- 1_{\pi ^{-1}(\pi (E)^\infty )}\Vert _{L^1(A_j)} \xrightarrow [] 0 \quad \text {as }t_k \xrightarrow [] 0. \end{aligned}$$

Hence, we can construct a locally finite open cover $\{A_j\}_{j \in \mathbb {N}}$ of $\mathbb {R}^n \setminus \{0\}$, such that for every $j \in \mathbb {N}$ condition (5) is satisfied. Setting $B_1:=A_1$ and $B_j:=A_j {\setminus } \cup _{i=1}^{j-1}B_i$ we obtain a refinement of the cover $\{A_j\}_{j \in \mathbb {N}}$ consisting of disjoint sets. Since $\{A_j\}_{j \in \mathbb {N}}$ is locally finite in $\mathbb {R}^n \setminus \{0\}$ also $\{B_j\}_{j \in \mathbb {N}}$ has this property. Hence, for every $r,\varepsilon >0$ with $r>\varepsilon$, there exists a finite subset $I_{r,\varepsilon } \subset \mathbb {N}$ such that $\{B_j\}_{j \in I_{r,\varepsilon }}$ covers $B_r(0) {\setminus } B_{\varepsilon }(0)$. Having fixed $r>\varepsilon >0$ we then obtain

6 $$\begin{aligned}&\Vert 1_{E/t_k}- 1_{\pi ^{-1}(\pi (E)^\infty )} \Vert _{L^1(B_r(0))} \nonumber \\&\le \Vert 1_{E/t_k}- 1_{\pi ^{-1}(\pi (E)^\infty )}\Vert _{L^1(B_\varepsilon (0))}+ \sum _{j \in I_{r,\varepsilon }} \Vert 1_{E/t_k}- 1_{\pi ^{-1}(\pi (E)^\infty )}\Vert _{L^1(B_j)}. \end{aligned}$$

Since $E/t_k$ and $\pi ^{-1}(\pi (E)^\infty )$ are both perimeter minimizing, it follows from Lemma 2.15 that

$$\begin{aligned} \sup _{k \in \mathbb {N}} \Vert 1_{E/t_k}- 1_{\pi ^{-1}(\pi (E)^\infty )}\Vert _{L^1(B_\varepsilon (0))} \le c(n) \mathcal {H}^n (B_\varepsilon (0)) \xrightarrow [] 0 \quad \text {as }\varepsilon \xrightarrow [] 0. \end{aligned}$$

On the other hand, since $I_{r,\varepsilon } \subset \mathbb {N}$ is finite, it holds

$$\begin{aligned} \sum _{j \in I_{r,\varepsilon }} \Vert 1_{E/t_k}- 1_{\pi ^{-1}(\pi (E)^\infty )}\Vert _{L^1(B_j)} \xrightarrow [] 0 \quad \text {as }t_k \xrightarrow [] 0. \end{aligned}$$

Hence, passing to the limit in (6), one obtains

$$\begin{aligned} \Vert 1_{E/t_k}- 1_{\pi ^{-1}(\pi (E)^\infty )}\Vert _{L^1(B_r(0))} \xrightarrow [] 0 \quad \text {as }t_k \xrightarrow [] 0, \end{aligned}$$

proving claim (4).

Since $\pi (E)^\infty \subset \mathbb {R}^n/\Gamma$ is a cone, $\pi ^{-1}(\pi (E)^\infty ) \subset \mathbb {R}^n$ is also a cone. Since $0 \in \partial (\pi (E)^\infty ) \subset \mathbb {R}^n/\Gamma$, then $0 \in \partial (\pi ^{-1}(\pi (E)^\infty )) \subset \mathbb {R}^n$ as well. Finally, since $\pi (E)^\infty \subset \mathbb {R}^n/\Gamma$ is a perimeter minimizer, $\pi ^{-1}(\pi (E)^\infty ) \subset \mathbb {R}^n$ is a $\Gamma$-symmetric set minimizing the perimeter against $\Gamma$-symmetric competitors by Lemma 3.5. $\square$

### Definition 3.8

($\Gamma$-symmetric functions) Given a $\Gamma$-symmetric set $E \subset \mathbb {R}^n$ we say that a function  is  for every .

### Lemma 3.9

Let $E \subset \mathbb {R}^n$ be a $\Gamma$-symmetric cone which is smooth in $\mathbb {R}^n \setminus \{0\}$ and has zero mean curvature in $\mathbb {R}^n \setminus \{0\}$. There exists a smooth $\Gamma$-symmetric extension of  to .

### Proof

Since *E* is a cone which is smooth in $\mathbb {R}^n \setminus \{0\}$, the intersection $S:=\partial E \cap S^{n-1}$ is a $n-2$ dimensional closed smooth manifold in $S^{n-1}$. We now consider the function $|\Pi _E|^2$ restricted to *S*. We show that it can be extended to a function  with the property that for every $g \in \Gamma$ and every $x \in S^{n-1}$ it holds $h(g \cdot x)=h(x)$.

Let *U* be a tubular neighborhood of *S* in $S^{n-1}$ and let $\pi _S:U \xrightarrow [] S$ be the nearest point projection in $S^{n-1}$. We define $h_1 \in C^\infty (U)$ by $h_1(x):=|\Pi _E|^2(\pi _S(x)).$ Let $\eta \in C^\infty _c(U)$ be a function on *U* which depends only on the distance from *S* and is identically equal to 1 on *S*. We define $h \in C^\infty (S^{n-1})$ by

$$\begin{aligned} h(x):= {\left\{ \begin{array}{ll} \eta (x) h_1(x) &  x \in U \\ 0 &  x \notin U. \end{array}\right. } \end{aligned}$$

Since $S \subset S^{n-1}$ is invariant under the action of $\Gamma$ on $S^{n-1}$, so is *U*. Similarly, the restricted function $|\Pi _E|^2 \in C^\infty (S)$ has the property that for every $g \in \Gamma$ and every $x \in S$ it holds $|\Pi _E|^2(g \cdot x)=|\Pi _E|^2(x)$. Consequently, the extension $h_1 \in C^\infty (U)$ is also invariant under the action of $\Gamma$ on *U*. The same is true for $h \in C^\infty (S^{n-1})$ given the choice of $\eta \in C^\infty _c(U)$.

Finally, we define the extension $f \in C^\infty (\mathbb {R}^n{\setminus } \{0\})$ in polar coordinates by $f(\omega ,r):=r^{-2}h(\omega )$. Since  is homogeneous of degree $-2$ by Remark 2.21, the function $f \in C^\infty (\mathbb {R}^n {\setminus } \{0\})$ is the desired extension. $\square$

We here prove Theorem 2. Using the results we have shown so far we are able to reduce ourselves to studying $\Gamma$-symmetric sets in $\mathbb {R}^n$ which minimize the perimeter with respect to $\Gamma$-symmetric competitors. We are then able to follow the computations of Simons from [45] to conclude that such sets are half spaces. We mention that a more detailed exposition of the same computations can also be found in [39, Theorem 10.10].

### Proof of Theorem 2

By a standard classification result regarding manifolds of constant sectional curvature (see, for instance, [33, Theorem 4.1]), *M* is isometric to $S^n / \Gamma$, where $\Gamma$ is a discrete group of isometries of $S^n$ acting freely. In conformity with the rest of this section, we consider the induced action of $\Gamma$ on $\mathbb {R}^{n+1}$. We refer to the associated projection as .

Let us point out that $C(S^n/\Gamma )$ is isometric to $\mathbb {R}^{n+1}/\Gamma$. Therefore, to prove the statement of the theorem it is sufficient to show the following: if $F \subset \mathbb {R}^{n+1}/\Gamma$ is a perimeter minimizing set such that $0 \in \partial F$, then $\Gamma =\{id_{S^n}\}$, and $F \subset \mathbb {R}^{n+1}$ is a half space.

By Lemma 3.7 there exists a $\Gamma$-symmetric cone $G \subset \mathbb {R}^{n+1}$ which minimizes the perimeter against $\Gamma$-symmetric competitors, whose boundary contains 0, and such that

$$\begin{aligned} (\mathbb {R}^{n+1},G,0) \in \textrm{Tan}(\mathbb {R}^{n+1}, \pi ^{-1}(F), 0). \end{aligned}$$

By Lemma 3.6 the cone *G* is smooth with vanishing mean curvature except at $\{0\}$. We follow the computations of [45] for perimeter minimizing cones in $\mathbb {R}^{n+1}$ to show that *G* is a half space.

If $g \in C^{\infty }_c(\mathbb {R}^{n+1} {\setminus } \{0\})$ is a $\Gamma$-symmetric function, then the set $G_t$ (using the notation of Lemma 2.22) is $\Gamma$-symmetric. In particular, this holds if $g \in C^{\infty }_c(\mathbb {R}^{n+1}{\setminus } \{0\})$ is a radial function. Let us denote by $|\Pi _G|^2$ the $\Gamma$-symmetric extension of  to  obtained in Lemma 3.9. Applying Lemma 2.22 to the $\Gamma$-symmetric product $g |\Pi _G|$, where  is a radial function, we have

$$\begin{aligned} \int _{\partial G} |\nabla _G (g |\Pi _G|)|^2-|\Pi _G|^2(g |\Pi _G|)^2 \, d\mathcal {H}^{n} \ge 0. \end{aligned}$$

Using Lemma 2.23 we obtain

$$\begin{aligned} \int _{\partial G} |\Pi _G|^4g^2 \, d\mathcal {H}^{n}&\le \int _{\partial G} \Big (|\Pi _G|^2 |\nabla _G g|^2+g^2 |\nabla _G |\Pi _G||^2 + \frac{1}{2} \nabla _G |\Pi _G|^2 \cdot \nabla _G g^2 \Big ) \, d\mathcal {H}^{n} \\&= \int _{\partial G} \Big (|\Pi _G|^2 |\nabla _G g|^2+g^2 |\nabla _G |\Pi _G||^2 - \frac{1}{2} g^2 \Delta _G |\Pi _G|^2 \Big ) \, d\mathcal {H}^{n}. \end{aligned}$$

From Lemma 2.24 it follows that

7 $$\begin{aligned} \int _{\partial G}\Big ( |\nabla _G g|^2-\frac{2g^2}{|x|^2} \Big )|\Pi _G|^2 \, d\mathcal {H}^{n} \ge 0. \end{aligned}$$

The same is true by approximation for every radial function $g \in C^{\infty }(\mathbb {R}^{n + 1})$ such that

$$\begin{aligned} \int _{\partial G}\frac{g^2}{|x|^2}|\Pi _G|^2 \, d\mathcal {H}^{n} < + \infty . \end{aligned}$$

Since $|\Pi _G|^2$ is homogeneous of degree $-2$ by Remark 2.21, the previous condition holds if $g \in C^\infty (\mathbb {R}^{n+1})$ satisfies

8 $$\begin{aligned} \int _{\partial G}\frac{g^2}{|x|^4}\, d\mathcal {H}^{n} < + \infty . \end{aligned}$$

A function $g \in C^\infty (\mathbb {R}^{n+1})$ of the form

$$\begin{aligned} g(x):=|x|^\alpha \max \{|x|,1\}^\beta \end{aligned}$$

satisfies (8) if

9 $$\begin{aligned} {\left\{ \begin{array}{ll} \alpha > \frac{4-n}{2}, \\ \alpha +\beta <\frac{4-n}{2}. \end{array}\right. } \end{aligned}$$

Plugging such *g* in (7) we obtain

10 $$\begin{aligned}  &   (\alpha ^2-2)\int _{\partial G \cap B_1(0)}|x|^{2\alpha -2}|\Pi _G|^2 \, d\mathcal {H}^n + ((\alpha +\beta )^2-2)\nonumber \\  &   \int _{\partial G \setminus B_1(0)} |x|^{2(\alpha +\beta )-2}|\Pi _G|^2 \, d \mathcal {H}^n \ge 0. \end{aligned}$$

Since $n \le 6$ we can choose $\alpha$ and $\beta$ compatible with (9) and such that $\alpha ^2-2 \le 0$ and $(\alpha +\beta )^2-2 \le 0$. With such choice of $\alpha$ and $\beta$, from inequality (10) it follows that $|\Pi _G|^2$ is identically 0 on $\partial G$. Since the second fundamental form of $\partial G$ in $\mathbb {R}^n$ vanishes, the outer unit normal vector to *G* is constant on $\partial G$. Hence, $G \subset \mathbb {R}^{n+1}$ is a half space.

Let us show that $\Gamma = \{id_{S^{n}}\}$. Since *G* is a half space, $\partial G \cap S^{n}=S^{n-1}$. Since *G* is $\Gamma$-symmetric, $\partial G \cap S^{n}$ is sent to itself by all elements of $\Gamma$. We claim that the poles with respect to $\partial G \cap S^{n}$ (that is, the two points on $S^{n}$ at maximal distance from $\partial G \cap S^{n}$) are swapped by every element of $\Gamma$ which is not the identity. Indeed, the poles cannot be fixed as the action of $\Gamma$ is free. Furthermore, the distance between each pole and $\partial G\cap S^{n}$ must be preserved since all elements of $\Gamma$ are isometries. In particular, all the elements of $\Gamma$ which are not the identity swap the poles.

Since $\Gamma$ acts on $\mathbb {R}^n$ isometrically, for every $g \in \Gamma$, $g \ne id_{S^n}$, there is a neighborhood $U \subset \mathbb {R}^n$ of one of the poles such that $U \subset G$ and $g \cdot U \subset \mathbb {R}^n {\setminus } G$. Consequently, any such element of $\Gamma$ cannot map the half space *G* to itself. As *G* is $\Gamma$-symmetric, we conclude that $\Gamma$ is trivial.

Finally, the initial set $F \subset \mathbb {R}^{n+1}/\Gamma \cong \mathbb {R}^{n+1}$ is a half space since there are no nontrivial perimeter minimizing sets in Euclidean spaces of dimension less than 8. $\square$

Theorem 2 fails if $n \ge 7$ as shown by the next example.

### Example 3.10

Let $n=7$ and let $\Gamma :=\{id_{S^7},-id_{S^7}\}$. Let $E \subset \mathbb {R}^8$ be the Simons cone, i.e.

$$\begin{aligned} E=\{|x_1|^2+|x_2|^2+|x_3|^2+|x_4|^2 > |x_5|^2+|x_6|^2+|x_7|^2+|x_8|^2 \} \subset \mathbb {R}^8. \end{aligned}$$

Let us note that $E \subset \mathbb {R}^{8}$ is a $\Gamma$-symmetric set which minimizes the perimeter (and, in particular, it minimizes the perimeter against $\Gamma$-symmetric competitors). By Lemma 3.5, $\pi (E) \subset \mathbb {R}^{8}/\Gamma \cong C(S^7/\Gamma )$ is a perimeter minimizing set. Moreover, the boundary of $\pi (E)$ contains 0.

Let now $n=7+k$ with $k \in \mathbb {N}$ and let $\Gamma :=\{id_{S^{7+k}},-id_{S^{k+7}}\}$. Let $E \times \mathbb {R}^k \subset \mathbb {R}^8 \times \mathbb {R}^k$ be the product of the Simons cone with the extra Euclidean factors. $E \times \mathbb {R}^k \subset \mathbb {R}^8 \times \mathbb {R}^k$ is a $\Gamma$-symmetric set which minimizes the perimeter (and, in particular, it minimizes the perimeter against $\Gamma$-symmetric competitors). By Lemma 3.5, $\pi (E) \subset \mathbb {R}^{8+k}/\Gamma \cong C(S^{7+k}/\Gamma )$ is a perimeter minimizing set. Moreover, the boundary of $\pi (E)$ contains 0.

Building on Theorem 2, we prove Theorem 1.

### Proof of Theorem 1

The proof is divided in two steps: we start by showing $\mathcal {S}^E \setminus \mathcal {S}^E_{n-4} = \emptyset$, and then prove $\mathcal {S}^E_{n-4} {\setminus } \mathcal {S}^E_{n-5} = \emptyset$.

Let us show $\mathcal {S}^E {\setminus } \mathcal {S}^E_{n-4} = \emptyset$. Suppose by contradiction that $x \in \mathcal {S}^E {\setminus } \mathcal {S}^E_{n-4}$. Then, there exists a pointed metric space $(Y,d_y,p)$ and a perimeter minimizing set of the form $\mathbb {R}^{n-3} \times A \subset \mathbb {R}^{n-3} \times Y$ whose boundary contains $(0,p) \in \mathbb {R}^{n-3} \times Y$ such that

$$\begin{aligned} (\mathbb {R}^{n-3} \times Y, \mathbb {R}^{n-3} \times A,(0,p)) \in \textrm{Tan}(X,E,x). \end{aligned}$$

By Lemma 2.19, $A \subset Y$ is a perimeter minimizing set whose boundary contains *p*. Moreover, $\mathbb {R}^{n-3} \times Y \cong \mathbb {R}^n$ by Theorem 2.5 since

$$\begin{aligned} (\mathbb {R}^{n-3} \times Y, (0,p)) \in \textrm{Tan}(X,x). \end{aligned}$$

Therefore, $A \subset \mathbb {R}^3$ is a half space as it minimizes the perimeter and has non-empty boundary. We conclude that $x \not \in \mathcal {S}^E {\setminus } \mathcal {S}^E_{n-4}$, a contradiction.

In the rest of the proof we show $\mathcal {S}^E_{n-4} \setminus \mathcal {S}^E_{n-5} = \emptyset$. Suppose by contradiction that there exists $x \in \mathcal {S}^E_{n -4} \setminus \mathcal {S}^E_{n-5}$.

By Theorem 2.8 the tangent space $\textrm{Tan}(X,x)$ has an element isometric to $\mathbb {R}^{n-4} \times C(S^3/\Gamma )$, where $\Gamma \subset O(4)$ is a discrete group acting freely. Hence,

$$\begin{aligned} (\mathbb {R}^{n-4} \times C(S^3/\Gamma ), \mathbb {R}^{n-4} \times F,(0,p)) \in \textrm{Tan}(X,E,x), \end{aligned}$$

where $p \in C(S^3/\Gamma )$ is the tip, and $\mathbb {R}^{n-4} \times F \subset \mathbb {R}^{n-4} \times C(S^3/\Gamma )$ is a perimeter minimizing set whose boundary contains $(0,p) \in \mathbb {R}^{n-4} \times C(S^3/\Gamma )$.

By Lemma 2.19, $F \subset C(S^3/\Gamma )$ is a perimeter minimizing set whose boundary contains *p*. By applying Theorem 2, we can then infer that $C(S^3/\Gamma )$ is isometric to $\mathbb {R}^4$ and that *F* is a half space, contradicting $x \in \mathcal {S}^E_{n-4} {\setminus } \mathcal {S}^E_{n-5}$. Therefore, $\mathcal {S}^E {\setminus } \mathcal {S}^E_{n-5} = \emptyset$ as claimed.

Since $\mathcal {S}^E=\mathcal {S}^E_{n-5}$, Theorem 2.18 implies that $\textrm{dim}_{\mathcal {H}}(\mathcal {S}^E) \le n-5.$ $\square$

Theorem 1 is sharp: as shown in the following example, there exists a noncollapsed limit of manifolds with two-sided bounds on the Ricci curvature with a perimeter minimizing set *E* such that $\mathcal {S}^E_{n-5}$ is non-empty.

### Example 3.11

The cone $C(\mathbb {R}\mathbb {P}^3)$, arising as the blow-down of the Eguchi-Hanson manifold (see [28, Example 2.15] for this construction; the Eguchi-Hanson metric was originally defined in [22, 36]), is a noncollapsed limit of manifolds with two-sided bounds on the Ricci curvature which is singular at the tip.

By a standard calibration argument it is possible to show that the set

$$\begin{aligned} C(\mathbb {R}\mathbb {P}^3) \times \mathbb {[}0,+\infty ) \subset C(\mathbb {R}\mathbb {P}^3) \times \mathbb {R} \end{aligned}$$

is a perimeter minimizing set. Moreover the tip of $C(\mathbb {R}\mathbb {P}^3)$ belongs to $\mathcal {S}^E_{n-5}$.

As mentioned in Remark 1.1, the regularity of a set $E \subset X$ satisfying the conditions of Theorem 1 can be improved substantially if we consider the restriction of $\partial E$ to the regular set $\mathcal {R}(X)$. The following proposition follows by repeating part of the proof of [15, Theorem A5], which in turn builds on a theorem of Allard [1, The Regularity Theorem p. 27] (which applies to minimizers of certain parametric integrands).

### Proposition 3.12

Let (*X*, *d*, *p*) be a noncollapsed limit of manifolds with two-sided bounds on the Ricci curvature of dimension *n*. Let $E \subset X$ be a locally perimeter minimizing set. The set $\partial E \cap \mathcal {R}(X)$ is a $C^{1,\alpha }$ hypersurface of $\mathcal {R}(X)$ for every $\alpha \in (0,1)$ outside of a closed set of Hausdorff dimension at most $n-8$.

### Proof

By [44, Theorem 6.23], it holds

$$\begin{aligned} \textrm{dim}_{\mathcal {H}}(\mathcal {S}^E \cap \mathcal {R}(X)) \le n-8. \end{aligned}$$

Let $\alpha \in (0,1)$ be fixed. By Theorem 2.7, $\mathcal {R}(X)$ is a $C^{1,\alpha }$ open manifold. To conclude, we fix $x_0 \in (\partial E {\setminus } \mathcal {S}^E) \cap \mathcal {R}(X)$ and we show that $\partial E$ is a $C^{1,\alpha }$ hypersurface in a neighbourhood of $x_0$.

Let $\epsilon >0$ be fixed. By Theorem 2.6, we can identify a neighbourhood of $x_0$ in *X* with the Euclidean ball $B_2(0^n) \subset \mathbb {R}^n$ equipped with a Riemannian metric *g* such that

11 $$\begin{aligned} \Vert g_{ij}-\delta _{ij}\Vert _{C^1(B_2(0^n))} < \epsilon . \end{aligned}$$

With this identification, $E \subset B_2(0^n)$ and $0^n \in \partial E {\setminus } \mathcal {S}^E$. Up to restricting to a smaller ball, we can also assume that *E* is perimeter minimizing in $B_2(0^n)$.

Let $\mathcal {H}_e^{n-1}$ denote the Euclidean $n-1$ dimensional Hausdorff measure in $B_2(0^n)$. By the area formula, the perimeter measure of *E* in $(B_2(0^n),g)$, denoted $P(E,\cdot )$, coincides with12where $Cof_x$ is the cofactor matrix of $(g_{ij})$ at *x*, $\nu _x$ is the distributional Euclidean unit normal to *E* at *x*, $\partial ^*E$ is the Euclidean reduced boundary of *E*, and $\langle \cdot ,\cdot \rangle$ is the Euclidean scalar product.

Since $0^n$ is a regular point for *E*, when one takes a blow up of $(B_2(0^n),g)$ in $0^n$, *E* converges to a half space *H*. It follows from [44, Theorem 2.42] that the perimeter of *E* converges weakly to the perimeter of *H*. Furthermore, $\partial E$ converges in the Kuratowski sense to the boundary of *H*. Let  be the projection map on the normal line to *H* in $\mathbb {R}^n$.

By the aforementioned convergence properties of $\partial E$, for every $\epsilon _1>0$, there exists $r \in (0,1)$ such that

13 $$\begin{aligned} 1-\epsilon _1 \le \Big | \frac{P(E,B_r(0^n))}{\omega _{n-1}r^{n-1}} \Big | \le 1+\epsilon _1, \quad |\pi (\partial E \cap B_{2r}(0^n))| \le \epsilon _1 r. \end{aligned}$$

It follows from (11), (13), the representation of the perimeter functional (12) and [1, The Regularity Theorem p. 27] that there exists a function $u \in C^{1,\alpha }(H \cap B_s(0^{n-1}))$ for a sufficiently small $s>0$, such that $\partial E \cap B_s(0^n)$ coincides with the graph of *u*. $\square$

## Data Availability

Not applicable.
